# Detection of a novel *PAX6* variant in a Chinese family with multiple ocular abnormalities

**DOI:** 10.1186/s12886-022-02256-7

**Published:** 2022-01-16

**Authors:** Junyi Ouyang, Ziyan Cai, Yinjie Guo, Fen Nie, Mengdan Cao, Xuanchu Duan

**Affiliations:** 1 Jinan University, Guangzhou, Guangdong China; 2 Aier School of Ophthalmology, Central South University, Changsha, Hunan China; 3 Aier Glaucoma Research Institute, Changsha Aier Eye Hospital, Changsha, Hunan China; 4 Department of Ophthalmology, The Second Xiangya Hospital, Central South University, Changsha, Hunan Province China

**Keywords:** PAX6, Aniridia, DNA variant, Phenotype

## Abstract

**Background:**

Aniridia is a congenital, panocular disease that can affect the cornea, anterior chamber angle, iris, lens, retina and optic nerve. *PAX6* loss-of-function variants are the most common cause of aniridia, and variants throughout the gene have been linked to a range of ophthalmic abnormalities. Furthermore, particular variants at a given site in *PAX6* lead to distinct phenotypes. This study aimed to characterize genetic variants associated with congenital aniridia in a Chinese family.

**Methods:**

The proband and family underwent ophthalmologic examinations. DNA was sampled from the peripheral blood of all 6 individuals, and whole-exome sequencing was performed. Sanger sequencing was used to verify the variant in this family members.

**Results:**

A novel variant (c.114_119delinsAATTTCC: p.Pro39llefsTer17) in the *PAX6* gene was identified in subjects II-1, III-1 and III-2, who exhibited complete aniridia and cataracts. The proband and the proband’s brother also had glaucoma, high myopia, and foveal hypoplasia.

**Conclusions:**

We identified that a novel *PAX6* frameshift heterozygous deletion variant is the predominant cause of aniridia in this Chinese family.

**Trial registration:**

We did not perform any health-related interventions for the participants.

**Supplementary Information:**

The online version contains supplementary material available at 10.1186/s12886-022-02256-7.

## Background

Aniridia is an eye disorder defined as partial or complete absence of the iris that can be congenital or caused by injury. Congenital aniridia is a sporadic [1] rare condition that affects 1:64,000–1:96,000 individuals, and up to two-thirds of patients exhibit an autosomal dominant form of the disorder [2].

Although the absence of the iris is the most prominent sign of this condition, congenital aniridia is also associated with abnormalities in the cornea, retina, lens, anterior chamber angle, and optic nerve. Most aniridia patients exhibit macular hypoplasia, nystagmus, and significant visual impairment, and a small subset have optic nerve hypoplasia [3]. In addition, patients with aniridia often develop a range of secondary ocular complications, including cataracts, aniridic keratopathy, and glaucoma. In fact, glaucoma affects up to 70% of aniridic patients [4].

Over 500 variants in the *PAX6* gene and its regulatory regions have been characterized to date. Many of these variants account for *PAX6* haploinsufficiency, which leads to significant ocular and systemic abnormalities [5]. In the present study, we describe a novel *PAX6* variant associated with congenital aniridia in a Chinese family.

## Methods

### Subjects and Clinical evaluation

Thorough ophthalmologic examinations were performed for the proband and her brother, including tests of visual acuity, intraocular pressure (IOP), slit-lamp analyses, anterior segment photography, visual field tests (Humphrey 750,Carl Zeiss, Germany), funduscopy, ultrasonic B analyses (Chiescan Quantel Medical, France), gonioscopic analyses, OCTA (optical coherence tomography angiography) assessments (RTVue-XR Avanti, v2017.1.0; OptoVue, Inc., CA, USA), and ultrasound biomicroscope (UBM) assessments (SW China). The proband’s other family members underwent a simple slit lamp examination.

### Variant screening and sequence analysis

#### Genomic DNA extraction

Approximately 4 ml of peripheral blood was sampled from the proband and her brother. Genomic DNA was extracted using a genomic DNA extraction and purification kit (TIANamp Blood DNA kit, #DP348–03) following the manufacturer’s protocol. The genomic DNA samples were stored at − 20 °C until use.

#### Library construction

1. Genome-wide library construction: DNA enzymatic fragmentation and genome-wide library construction were carried out using the DNA library construction kit of YEASEN Biology Company (Hieff NGS® OnePot DNA Library Prep Kit for Illumina®, YEASEN).
2. Construction of the clinical whole-exome capture library: a XGen Exome Research Panel V1.0 (Integrated DNA Technologies, Inc., USA) of the IDT company was used for capture and to construct the library of the proband and brother.

#### Clinical whole-exome sequencing

Paired-end sequencing was performed using the Illumina (San Diego, ca) sequencing platform with PE 150 patterns.

#### Bioinformatics analysis

Raw reads of low quality were removed, and the remaining reads were mapped to the UCSC (University of California Santa Cruz) hg19 reference genome (http://genome.ucsc.edu/). Single-nucleotide variations (SNVs) and insertion-deletion (InDel) variants were detected using the HaplotypeCaller function of Genome Analysis ToolKit (GATK, http://software.broadinstitute.org/gatk/).These annotated variants were then filtered based on the Annovar (http://www.openbioinformatics.org/annovar/) database. The databases used for pathogenicity prediction were SIFT (http://sift.jcvi.org), Polyphen2_HDIV (http://genetics.bwh.harvard.edu/pph2), Polyphen2_HVAR (http://genetics.bwh.harvard.edu/pph2), LRT (http://www.genetics.wustl.edu/jflab/lrt_query.html), variantTaster (http://www.varianttaster.org/), variantAssessor (http://variantassessor.org/r3/), FATHMM (http://fathmm.biocompute.org.uk), PROVEAN (http://provean.jcvi.org/index.php), MetaSVM (https://omictools.com/meta-svmtool), MetaLR (http://www.ensembl.info/tag/metalr/), M-CAP (http://bejerano.stanford.edu/mcap/), fathmm-MKL_coding (http://fathmm.biocompute.org.uk/fathmmMKL.htm). Quality control requirements: data volume > =6GB,average coverage> = 150X,30X coverage> = 98.5%,Q30 Qualification rate (%) > =89.17%).

#### Variant pathogenicity analysis

The guidelines of the American College of Medical Genetics and Genomics (ACMG) were used to facilitate appropriate data analysis (Table 3). Only genetic variations with known, definitive genetic associations were analysed. Genes with unknown pathogenicity or functionality were omitted from these analyses. In addition, common benign polymorphic variants, synonymous variants, and intronic variants not altering mRNA splicing were not included unless they have previously been reported in the literature as being pathogenic or were present in the database.

#### Variant verification

**Fig. 1:**
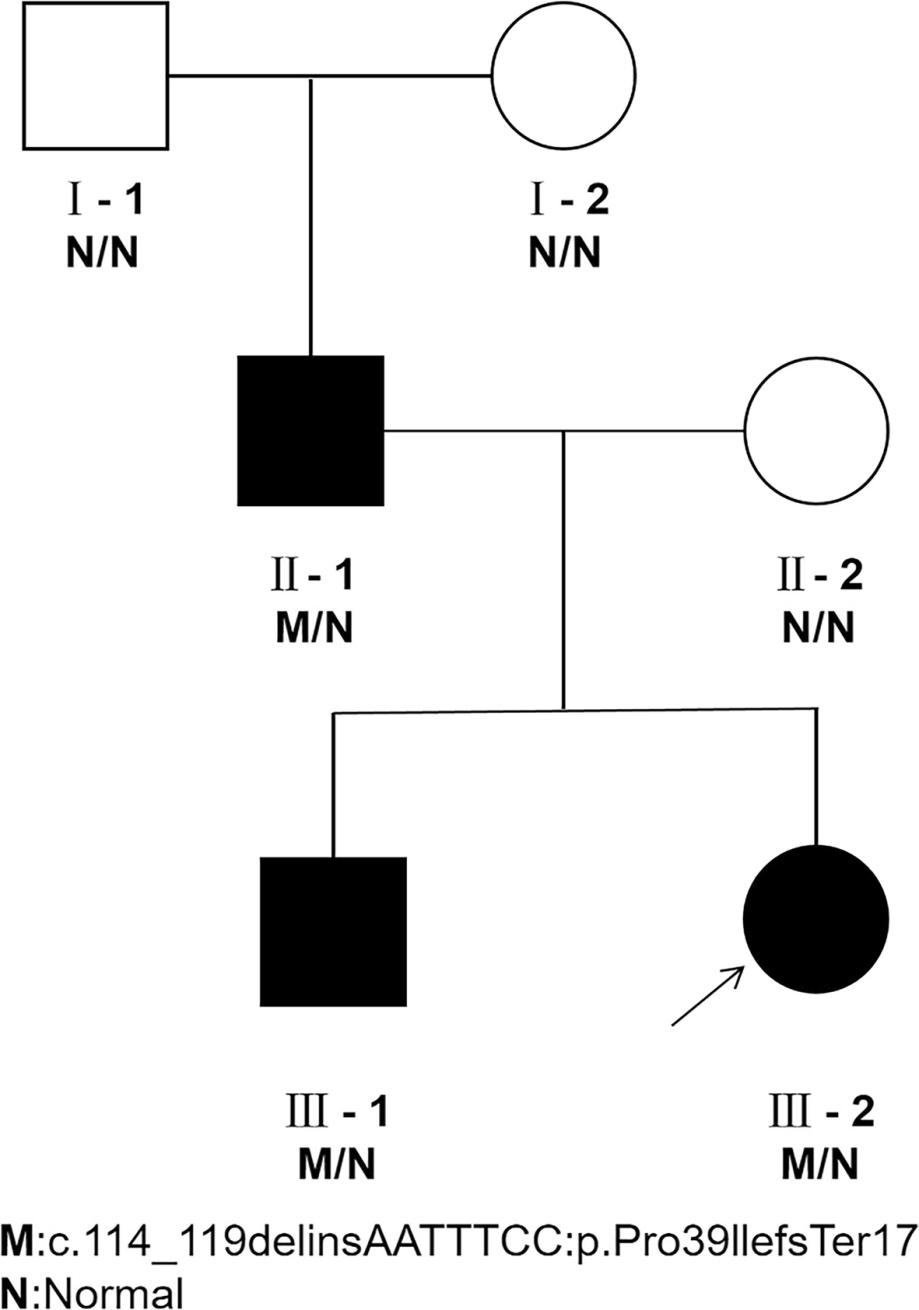
Pedigree of a Chinese family with aniridia. Squares and circles correspond to males and females, respectively. Black and white shapes correspond to affected and unaffected individuals, respectively. The proband is indicated with an arrow

Detected variations were validated by Sanger sequencing in the Chinese family. Primer3Plus (http://www.primer3plus.com/cgi-bin/dev/primer3plus.cgi) was used to design primers for *PAX6* gene c.114_119delinsAATTTCC: p.Pro39llefsTer17, and in-silico PCR (http://genome.ucsc.edu/cgi-bin/hgPcr) was used to verify primer specificity (Table 1). PCR amplification products for family members were sequenced using an ABI3730s AUTOMATIC DNA sequence Analyser (3730 DNA Analyser), and the results were analysed and compared using CodonCode Aligner software (CodonCode Corporation, USA) (Fig. 1).

**Table 1:** Sequencing primer details

Primer Name	Sequence	Amplified fragment length (bp)	Amplification reaction conditions.
SG2560_F	5′- TACAGTAAGAAATGAAGAGAGGGCGTT − 3’	499	3 min at 95 °C for PRE-denaturation,30 s at 95 °C for denaturation,30 s at 60 °C for primer annealing, 40 s at 72 °C for primer extension,
SG 2560_R	5′- GGGCACGGTTGCTTGGACT − 3’		30 cycles,and another 5 min at 72 °C for primer extension.

## Results

### Clinical data

#### The proband

**Fig. 2:**
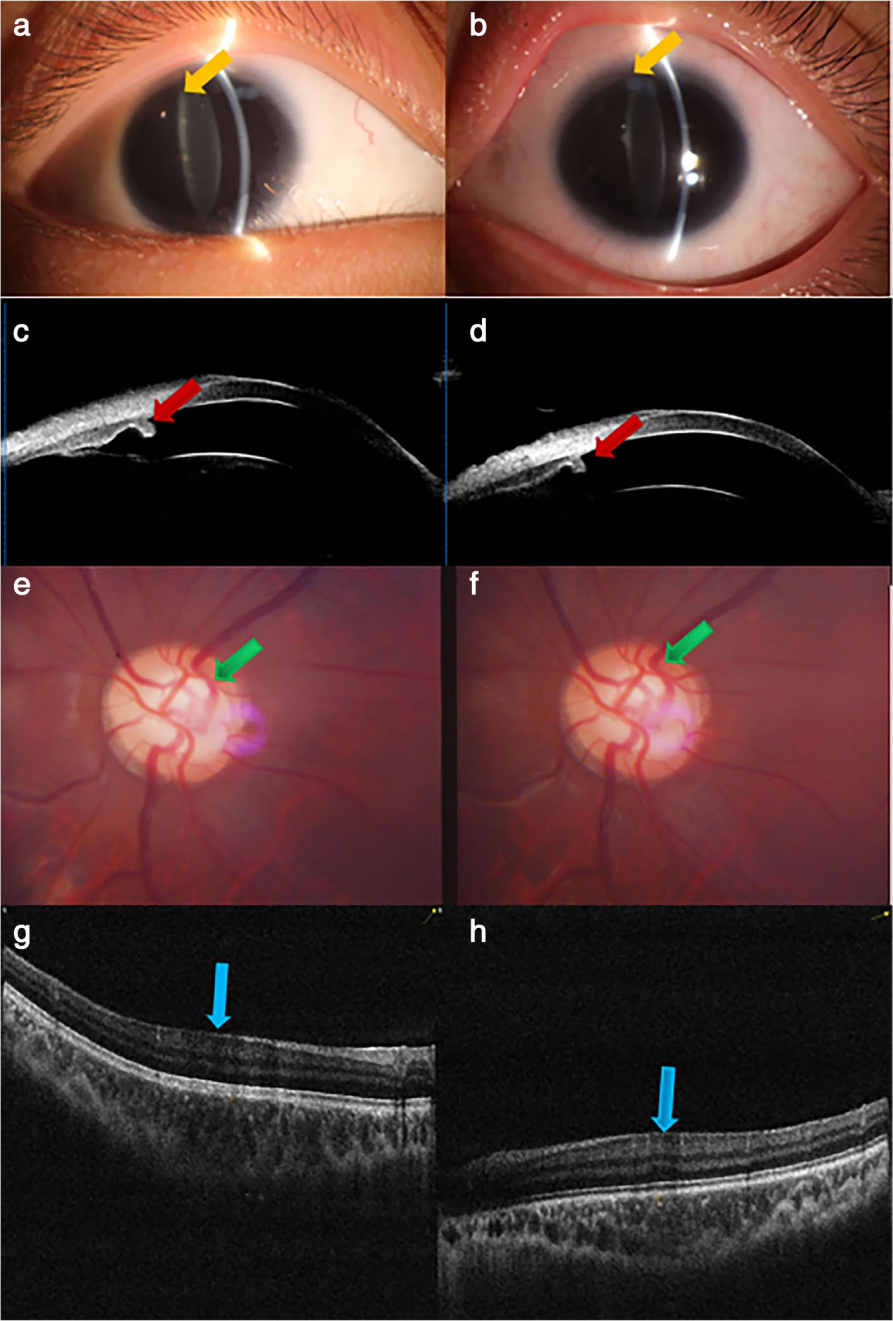
Clinical findings for the proband. The right and left columns correspond to the left and right eyes, respectively. Images of the anterior segment (**a**, **b**) and horizontally scanned UBM images of the anterior chamber (**c**, **d**). Images of the optic disc (**e**, **f**) and macular OCT examination (**g**, **h**). The yellow and red arrows indicated the residual image of iris. The blue arrows indicated the structure of fovea of macula. The green arrow indicated the structure of optic disc

A 13-year-old girl presented to our hospital complaining of bilateral blurred vision with no history of surgery or medical treatment of either eye. Her IOPs were 44 mmHg OD and 38 mmHg OS, as detected by Goldmann tonometry; her best-corrected visual acuity (BCVA) was 20/100 OD and 20/125 OS. Refractive errors were − 8.5 D OD and-6.0 D OS, with eyeball axis length (AL) values of 26.3 mm OD and 26.0 mm OS. The anterior chamber in both eyes appeared normal, and peripheral angles in both eyes were open. A slit-lamp examination showed the presence of bilateral peripheral cataracts and posterior capsular opacification, and UBM examination revealed iris coloboma. Further fundus examination revealed large optic disc with bilateral glaucomatous cupping and peripapillary atrophy. OCTA examination indicated diffuse superior and inferior RNFL (retinal nerve fibre layer) thinning, reduced wiVD (whole-image vessel density), idVD (inside disc vessel density), ppVD (peripapillary vessel density) vessel density, and significant foveal hypoplasia (Table 2). Visual field tests highlighted bilateral glaucomatous defects (Fig. 2). The proband had no other discomfort, particularly hearing loss or abnormal olfaction.

**Table 2:** RNFL, wiVD (whole-image vessel density), idVD (inside disc vessel density), and ppVD (peripapillary vessel density) values for the proband and the proband’s brother

Patient	RNFL(μm)		wiVD%		idVD%		PPVD%	
eye	OD	OS	OD	OS	OD	OS	OD	OS
proband	78	80	43.6	39.5	32.8	40.2	41.7	41.8
proband ‘s brother	85	82	42.3	40.6	43.8	33.4	43.4	42.8

### The brother of the proband

The 23-year-old brother of the proband reported a history of glaucoma that had been diagnosed one year prior in a different hospital, and he had been using IOP-lowering eye drops since.

**Fig. 3:**
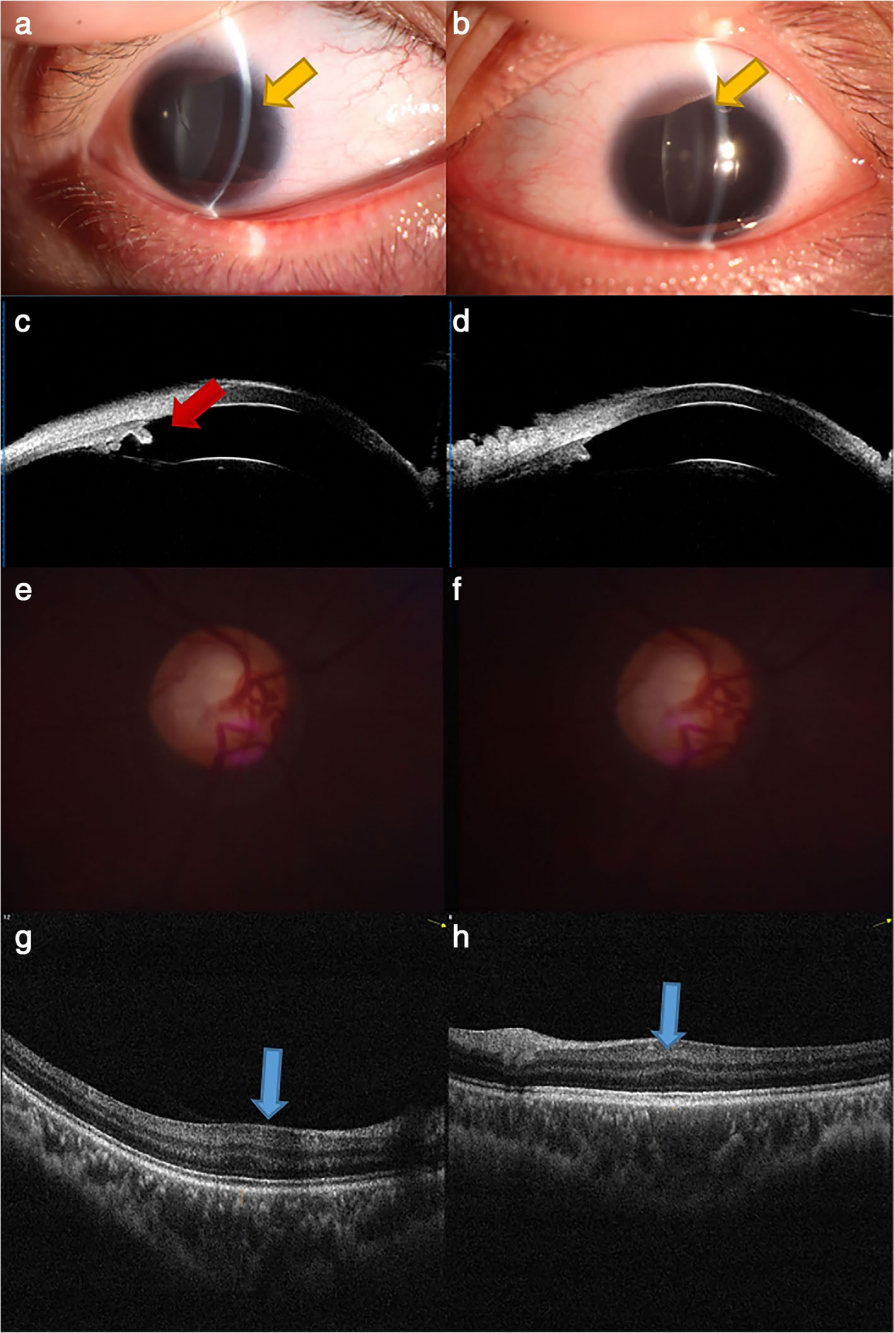
Clinical findings in the proband’s brother. The right and left columns correspond to the left and right eyes, respectively. Images of the anterior segment (**a**, **b**) and horizontally scanned UBM images of the anterior chamber (**c**, **d**). Images of the optic disc (**e**, **f**) and macular OCT examination (**g**, **h**). The yellow and red arrows indicated the residual image of iris. The blue arrows indicated the structure of fovea of macula. The green arrow indicated the structure of optic disc

His BCVA in both eyes was 20/80, with refractive error values of − 9.5 D OD and-10.25 D OS; eyeball AL values were 26.7 mm OD and 26.5 mm OS. He exhibited many of the same ophthalmic abnormalities as his sister, including complete aniridia, cataracts, glaucoma, high myopia, and foveal hypoplasia. In addition, the brother showed a decreased VD compared to the usual observation for healthy eyes. The superior and inferior RNFL of the brother’s eyes were thicker than that of his sister’s eyes (Table 2), and he had fewer pronounced bilateral glaucomatous visual field defects (Fig. 3). The proband’s brother also had no hearing loss or abnormal olfaction.

### Other family members

The proband’s father had limited vision, such as light perception, and a simple examination showed severe cataracts and complete aniridia. The proband’s mother and grandparents had no obvious eye problems.

#### Variant analysis

**Fig. 4:**
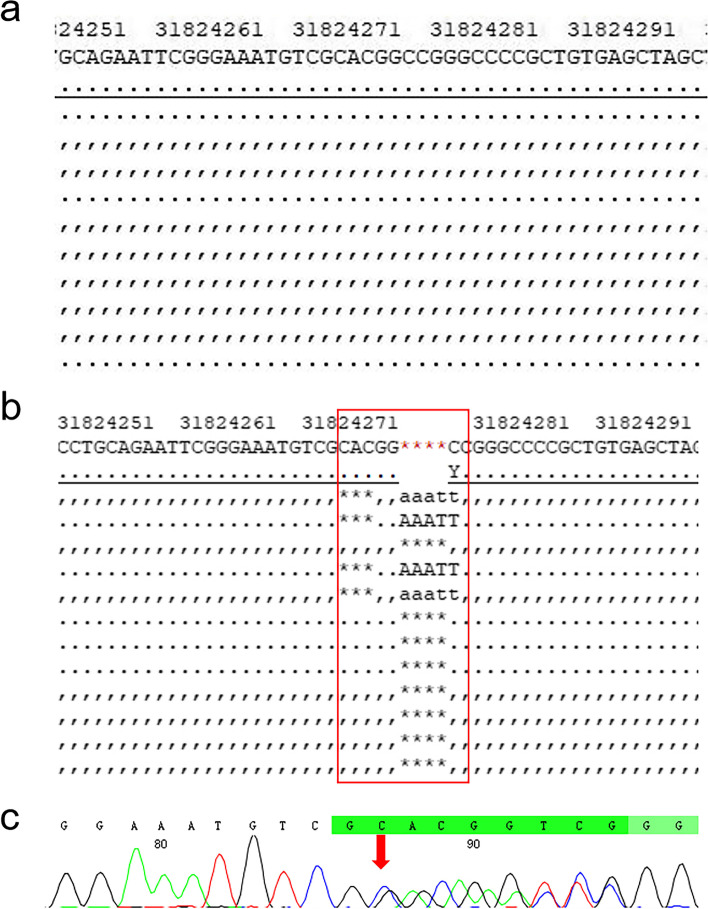
Sequence chromatograms showing the wild-type (**a**) and mutated (**b**) *PAX6* gene. The red box demonstrates the presence of a heterozygous frameshift deletion variant (c.114_119delinsAATTTCC: p.Pro39llefsTer17) in exon 5 of the *PAX6* gene. The red arrows indicate the site of the variant, with the dual peaks corresponding to the presence of the indicated heterozygous insertion variants

Next-generation sequencing analyses demonstrated the presence of a heterozygous frameshift deletion variant (c.114_119delinsAATTTCC: p.Pro39llefsTer17) in exon 5 of the *PAX6* gene. This variant, consisting of a 6-bp deletion and a 7-bp insertion, results in a frameshift from the 39th proline codon, resulting in the generation of a premature stop codon (Fig. 4). Based ACMG, the variant is a pathogenic variant (Table 3).

**Table 3:** The classification refers to ACMG guidelines

ACMG	Description of evidence	Classification results
PVS1	The pax6 gene in the clingen database (https://clinicalgenome.org/) was recorded with a single dose sensitive gene with a score of 3 .	Pathogenic variation
PM2_Supporting	It is a rare variant, not included in the Genome Aggregation Database (gnomAD,https://gnomad.broadinstitute.org/about) East Asian database.	
PP4	The patient’s clinical symptoms and family history were anastomotic with PAX6 gene abnormality.	

## Discussion

By analysing a Chinese family with a history of congenital aniridia, we identified a novel hybrid variant (c.114_119delinsAATTTCC: p.Pro39llefsTer17) in the *PAX6* gene. This variant comprises a 6-bp deletion and a 7-bp insertion resulting in premature truncation of the PAX6 protein. The affected brother and sister exhibited shared ophthalmic abnormalities, including cataracts, nystagmus, glaucoma, aniridia, and macular fovea hypoplasia. The *PAX6* gene, located on chromosome 11p13, was first characterized by Ton et al. in 1991 [6]. *PAX6* encodes a transcriptional regulator that is important for the development of organs and tissues, including the eyes. *PAX6* expression is detectable in the iris, lens, optic disc, corneal epithelium, ciliary body, retinal neuroepithelium, and retinal pigment epithelium. In 2005, Tzoulaki et al. characterized human *PAX6* variants and found that variants throughout the gene were associated with aniridia and related phenotypes [7]. In a study of 95 Chinese patients with aniridia, You et al. found *PAX6* loss-of-function variants to be the most common cause of aniridia [8]. The *PAX6* variant identified in these siblings in the present study (c.114_119delinsAATTTCC: p.Pro39llefsTer17) causes a frameshift from the 39th codon, leading to a premature stop codon. In light of prior studies, we hypothesize that this variant is likely to be the primary cause of the aniridia and other observed ophthalmic abnormalities in these siblings.

In their prior study of 95 Chinese aniridia patients, You et al. identified 47 different variants associated with the aniridia phenotype, including 6 frameshift InDel variants, 12 nonsense variants, 2 missense variants, 1 run-on variant, 1 synonymous variant, and 15 variants that altered mRNA splicing [8]. The human gene variant database (HGMD) currently includes 479 pathogenic *PAX6* variants (http://www.hgmd.cf.ac.uk/ac/gene.php?gene=PAX6). In total, 20 reports to date have described cases of patients with both insertion and deletion variants in *PAX6,* and such combination variants are likely to be associated with serious ophthalmic abnormalities. Our observations of abnormalities, including aniridia and glaucoma, in the patients in the present study are thus consistent with these prior studies.

The PAX6 protein is composed of four domains: two DNA-binding domains, including an N-terminal 128-amino acid paired box domain (PD) and a 61-amino acid homeodomain (HD), as well as a 79-amino acid glycine-rich hinge region and a C-terminal proline-rich serine transactivation domain [9, 10]. variants throughout *PAX6* have been linked to a range of ophthalmic abnormalities, with particular variants at a given site leading to distinct phenotypes. In the present subjects, glaucoma manifested at an earlier age and was more severe in the proband than in her brother. Two primary models have been proposed to describe the penetrance of *PAX6* variants. Dominant-negative *PAX6* variants are thought to enhance PAX6 binding to DNA, leading to abnormal dominant-negative effects as a result of premature PAX6 truncation [11]. Other *PAX6* variants are better described by a dose-effect model in which premature termination codons (PTCs) within the open reading frame lead to premature protein truncation as a result of nonsense-mediated mRNA decay (NMD). In such a dose-effect model, a single wild-type allele of *PAX6* is insufficient to ensure normal ocular development, leading to ophthalmic abnormalities [12]. Subtle phenotypic differences between patients with various *PAX6* variants may thus be attributable to slight differences in intracellular PAX6 levels [13]. In the present study, we identified a novel heterozygous frameshift variant in *PAX6* that results in a frameshift from the 39th proline codon and the generation of a premature stop codon. This variant begins in exon 5 in the PD domain and leads to truncation of the LNK (linker, glycine-rich hinge region), HD, and PST domains of the protein, resulting in a shortened polypeptide that is unlikely to be functional [12]. Haploinsufficiency is likely to explain the observed aniridia phenotype in the subjects of the present study, though the mechanistic link between genotype and phenotype in these patients needs to be fully characterized in future studies.

## Conclusions

In summary, we identified the novel heterozygous c.114_119delinsAATTTCC: p.Pro39llefsTer17 variant of the *PAX6* gene as a putative cause of aniridia in a Chinese family. These results expand the spectrum of known variants that cause *PAX6*-triggered congenital aniridia and enhance our understanding of the genetic aetiology of this condition. Furthermore, our findings may aid in the genetic diagnosis of aniridia.

## Supplementary Information

**Additional file 1.**

## Data Availability

All data generated or analysed during this study are included in this published article [and its supplementary information files].

## Abbreviations

IOP: Intraocular pressure
OCTA: Optical coherence tomography angiography
UBM: Ultrasound biomicroscope
SNVs: Single nucleotide variations
InDel: Insertion-deletion
GATK: Genome Analysis ToolKit
ACMG: The guidelines of the American College of Medical Genetics and Genomics
BCVA: Best-corrected visual acuity
RNFL: Retinal nerve fibre layer) thinning
wiVD: Whole image vessel density
idVD: Inside disc vessel density
ppVD: Peripapillary vessel density
VD: Vessel density
HGMD: The human gene variant database
PD: Paired box domain
HD: Homeodomain
NMD: Nonsense-mediated mRNA decay
PTCs: Premature termination codon
