# Supplementary material for: Detection of a novel PAX6 variant in a Chinese family with multiple ocular abnormalities
Source: BMC Ophthalmol. 2022 Jan 16;22:28. doi: 10.1186/s12886-022-02256-7 (PMC8761350; doi:10.1186/s12886-022-02256-7)

**Detection of a novel *PAX6*** **mutation in a Chinese family with multiple ocular abnormalities**

Junyi Ouyang^1,3,4^, Ziyan Cai^2^, Yinjie Guo^2^, Fen Nie^2^, Mengdan Cao^2^, Xuanchu Duan^1,3,4*^

1.Jinan University,Guangzhou,Guangdong,China.

2.Department of Ophthalmology, The Second Xiangya Hospital, Central South University, Changsha, Hunan Province, China.

3.Aier School of Ophthalmology, Central South University, Changsha, Hunan, China.

4.Aier Glaucoma Research Institute, Changsha Aier Eye Hospital, Changsha, Hunan, China.

*Correspondence author: Xuanchu Duan M.D., Ph.D. Jinan University, Guangzhou, Guangdong,China;Aier School of Ophthalmology, Central South University, Changsha, Hunan, China; Glaucoma Research Institute, Changsha Aier Eye Hospital, Changsha, Hunan, China. Email:[duanxchu@126.com](mailto:duanxchu@126.com)

**The result of Next Generation Sequencing**

Proband


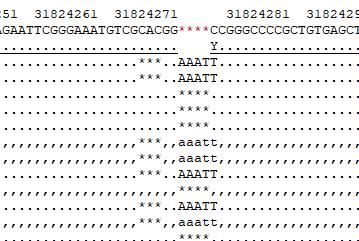


Proband’s father


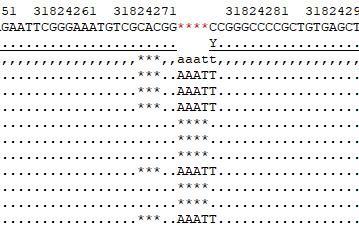


Proband’s mather


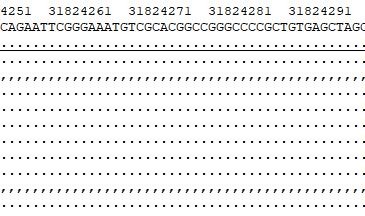


Proband’s grandfather


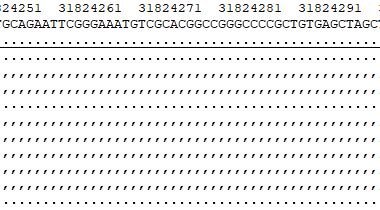


Proband’s grandmather


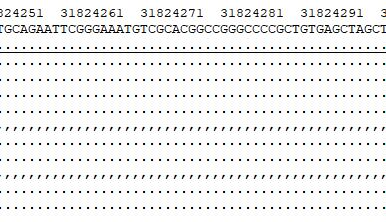


Proband’s brother


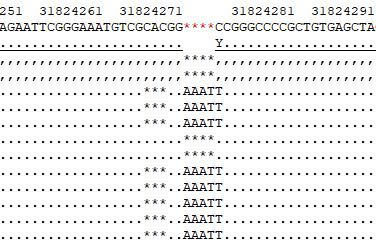

Supplement: Supplementary file 1 — Additional file 1. [file 12886_2022_2256_MOESM1_ESM.doc]
